# Fabrication of Antireflection Micro/Nanostructures on the Surface of Aluminum Alloy by Femtosecond Laser

**DOI:** 10.3390/mi12111406

**Published:** 2021-11-16

**Authors:** Mengdan Du, Quanquan Sun, Wei Jiao, Lifeng Shen, Xiao Chen, Junfeng Xiao, Jianfeng Xu

**Affiliations:** 1 State Key Laboratory of Digital Manufacturing Equipment and Technology, School of Mechanical Science and Engineering, Huazhong University of Science and Technology, Wuhan 430074, China; mddu@hust.edu.cn (M.D.); jiaowei6@huawei.com (W.J.); xiaochen@hust.edu.cn (X.C.); xiaojf@hust.edu.cn (J.X.); 2 Shanghai Aerospace Control Technology Institute, Shanghai 201109, China; sunquanquan1992@163.com (Q.S.); fdchrist@126.com (L.S.)

**Keywords:** femtosecond laser, aluminum alloy, antireflection, surface micro/nanostructure

## Abstract

Designed micro-nano structures on the surface of aluminum alloy provide excellent light trapping properties that can be used extensively in thermal photovoltaics, sensors, etc. However, the fabrication of high-performance antireflective micro-nano structures on aluminum alloy is challenging because aluminum has shallow intrinsic losses and weak absorption. A two-step strategy is proposed for fabricating broadband antireflection structures by superimposing nanostructures onto microscale structures. By optimizing the processing parameters of femtosecond laser, the average reflectances of 2.6% within the visible spectral region (400–800 nm) and 5.14% within the Vis-NIR spectral region (400–2500 nm) are obtained.

## 1. Introduction

Surface morphology is crucial in controlling the optical, chemical, biological, and other properties of solid surfaces [1]. Specially designed micro/nanostructures are utilized to tune reflectance of the surface, which offers opportunities in photovoltaics, sensors, etc. [2]. Traditionally, antireflection structures are fabricated by coating black paint on the surface. On the other hand, vertically aligned carbon nanotubes (VACNTs) are grown on the surface of workpiece in order to reduce effective refractive index to nearly one [3,4]. Carbon nanotubes (CNTs) on aluminum surfaces yield an efficient absorption rate in a range from ultraviolet to terahertz [5]. However, these CNTs are fragile in extreme environments and require controlled fabrication conditions [6]. Coating or deposition of another material on a surface tends to delaminate due to mismatching thermal expansion coefficients together with insufficient bonding, and its performance will decline over time [7]. In comparison, micro/nanostructures of the same material with substrates offer better stability and durability [2].

Femtosecond laser as a direct writing technology is a versatile fabrication method for micro/nanostructures that offers the following advantages: the capability to process almost all materials; the capability to induce nanostructures during ablating microstructures [8]; the capability to process nonplanar samples; and low requirements on the fabrication environment. Currently, there are many reports on antireflection micro/nanostructures prepared by the ultrafast laser on metal surfaces. The picosecond laser was used to prepare porous coral-like structures on copper, resulting in over 97% absorptivity in the visible spectral region and an average absorbance of 90% in the UV-vis-NIR regions (250–2500 nm) [8]. Coral-like microstructures were also prepared on the surface of NiTi alloy, which could achieve about 90% absorption from the UV to MIR region [9]. The cauliflower-shaped hierarchical surface micro/nanostructures were induced on copper by femtosecond laser. The average absorbance was 98% over a band of 200–800 nm [10]. In addition to micro-nano structures prepared with ultrafast laser, high-quality oxidized nanowires were uniformly grown on copper surfaces in order to enhance optical phonon dissipation. The structures achieved an ultralow reflectance of 0.6% at the infrared wavelength of 17 μm and stably kept at less than 3% within the wavelength range of 14–18 μm [11]. Luo et al. [12] adopted a two-step manufacturing strategy that combines the femtosecond laser with the nanosecond laser. They fabricated micropillars of different heights by adjusting the process repeat number of the femtosecond laser. Then, nanoparticles of different numbers and sizes were fabricated by changing the scanning speed of the nanosecond laser. A processing method of high and low speed hybrid scanning was proposed, which could apply to Cu, Ti, and W surfaces, via uniting multiple fast laser scanning to obtain microscale frame structures with single slow laser scanning to induce nanoscale features [13]. However, a super-blackening antireflection surface on the surface of aluminum alloy fabricated by pulsed laser has rarely been reported.

Aluminum is the only material with high reflectivity from the UV to IR region [14]. It is challenging to prepare antireflection surfaces of aluminum alloy due to its low melting temperature and high thermal conductivity [15]. As a kind of aluminum alloy with high-strength and high hardness in the Al-Cu-Mg series, aluminum alloy (2A12) is mainly used in various high-load parts and components [16], and it is more challenging to fabricate blackened aluminum alloy with higher hardness than compared pure aluminum. Antireflection micro-nano structures are mostly reported on metals such as Cu [2,8,10,11,12,13,17,18], Ti [12,13,18], Ti alloy [19,20], and Al [18,21,22,23]. There are few reports on preparing antireflection structures of aluminum alloy [12], especially on a broader spectrum (400–2500 nm) with high antireflection. In this paper, a two-step superimposing strategy was developed in order to process robust metal micro-nano structures via superimposing nanoscale structures onto microscale structures directly.

## 2. Experiment and Material

### 2.1. Material

The experimental material is aluminum alloy (2A12), and its chemical composition is provided in Table 1. The aluminum alloy was cut into a 50 mm × 50 mm × 2 mm sample, and the samples were polished to achieve surface roughness of 70 nm Ra. The workpiece was cleaned ultrasonically with ethanol for 3 min before laser ablation.

### 2.2. Experimental Device

Surface micro-nano structures on aluminum alloy surfaces were fabricated by an HR-Femto 50 femtosecond laser system (Huaray, HR-Femto-IR-80-60, Wuhan, China), which can generate 350 fs pulses at a central wavelength of 1035 nm and a repetition rate of 1 MHz. The maximum average power is 60 W. An x-y galvo is used to scan the laser beam on the aluminum alloy surface in an atmospheric environment.

### 2.3. Machining Parameters

**Figure 1:**
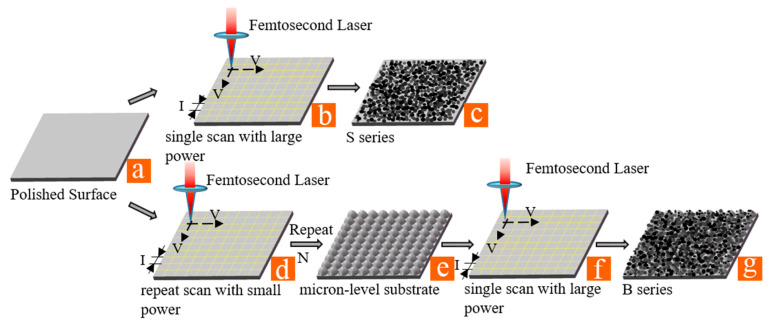
Schematic illustrations of two-step superimposing strategy: (**a**) polished surface, (**b**) single scan with large power, (**c**) S series structures, (**d**) repeat scan with small power, (**e**) micron-level substrate structures, (**f**) single scan with large power, and (**g**) B series structures.

Several laser processing parameters (illustrated in Figure 1) are involved during laser ablation, including pulse energy (J), power (P), laser fluence (F), scanning speed (V), scanning interval (I), scanning repetition (N), and defocusing (D). Two different structures (structure S and structure B) were prepared by using the HR-Femto 50 femtosecond laser system with specific laser processing parameters shown in Table 2. As shown in Figure 1c, the irregular surfaces with disorderly micro-nano particles and flocs were processed by the same parameters, represented by S. As shown in Figure 1g, the microscale structures were covered with disorderly micro-nano particles and flocs, which were processed by two kinds of parameters, denoted as B.

### 2.4. Characterization

After laser processing, the samples were ultrasonically cleaned with ethanol again. The micro-nano structure morphology of the sample surface was analyzed by a scanning electron microscope (FEI, Helios NanoLab G3 CX, Hillsboro, OR, USA). A white light interferometer (Zygo NewView 9000, Middlefield, CT, USA) was used to obtain the three-dimensional (3D) topography of the sample surface. A UV-Vis-NIR spectrophotometer with an integrating sphere (Shimadzu, SolidSpec-3700, Kyoto, Japan) was utilized in order to investigate their absorption behaviors in Vis-NIR regions. An energy spectrum analyzer (AMETEK EDAX, Mahwah, NJ, USA) was used to analyze the elements contained in the sample.

## 3. Results and Discussions

**Figure 2:**
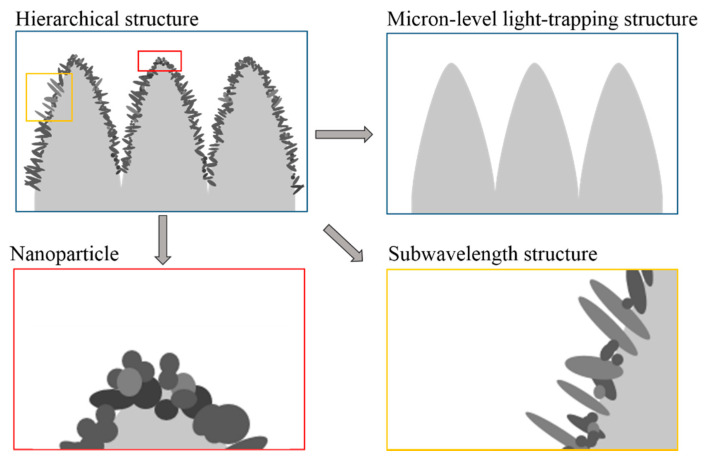
Schematic illustrations of hierarchical structure.

The reflectance depends on the structures’ depth, shape, and period related to the processing parameters [24]. The antireflection structures are hierarchical structures of different scales as shown in Figure 2.

### 3.1. Hierarchical Structure

(1) Microscale light-trapping structure

When the size of the cavity or the interval between the microstructures (usually referred to a size range from a few micrometers to tens of micrometers) is much larger than the wavelength of the incident light, the microcavity has excellent light-trapping property. The electromagnetic waves are trapped in the microcavity and reflected multiple times [13,25]; thus, their energy is absorbed. The optical properties of the structures do not depend on the wavelength of the incident light [17].

(2) Subwavelength structure

Subwavelength structures (referred to the structure for which its characteristic size is equivalent to or smaller than the working wavelength) promote grating coupling and cavity-trapping mechanisms by exciting surface plasmon polaritons (SPPs) [26]. Surface plasmon polaritons are divided into propagating surface plasmon polaritons (PSPPs) [27,28,29] and localized surface plasmons polaritons (LSPPs) [3,30,31]. Both PSPPs and LSPPs have good subwavelength properties and characteristics of bounding electromagnetic fields [32].

(3) Nanoparticle

Metallic nanoparticles (usually referred to a size range from a few nanometers to hundreds of nanometers) are excellent structures for exciting plasmon resonance and tuning the optical response of the surface [33]. Metal nanoparticles enhance light-trapping effects due to induced scattering by surface plasmon resonances [7]. Resonance and light extinction may occur at specific wavelength positions, and the size of the particle on the surface determines the total resonance frequency [2]. The aggregation of nanostructures results in the broadening effect of the resonance band and enhances broadband absorption, especially in the shortwave spectrum [8].

(4) Metal oxides layer

**Figure 3:**
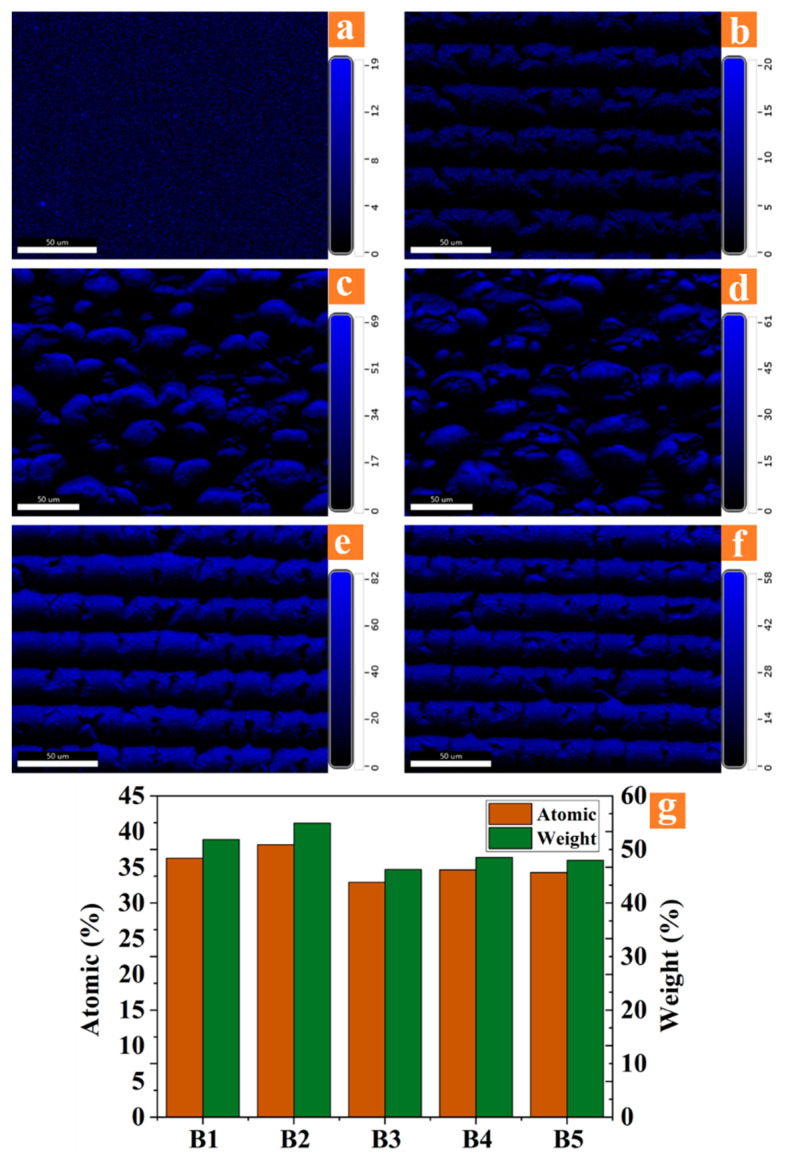
(**a**) Oxygen distribution on the polished surface; (**b**–**f**) oxygen distribution on the surface of B1–B5. (**g**) The normalized atomic percentage and mass percentage of oxygen on the surface of B1–B5.

Metal was oxidized on the surface of the microstructure as well as nanoparticles. The impedance mismatch caused by the vast refractive index difference between metal and air causes high reflectivity on the interface. Metal oxides as the transition layer can reduce impedance mismatch on the interface [34]. As observed in Figure 3, there is about 40% oxygen on the surface of the B series, which indicates that there are abundant metal oxides formed on the surface of aluminum alloy under femtosecond laser ablation. Metal oxides improve the antireflection performance of micro-nano structures by introducing a transitional medium between the metal and free space [11].

### 3.2. Antireflection Structure in the Visible Spectral Region: Subwavelength Structure + Nanoparticle

**Figure 4:**
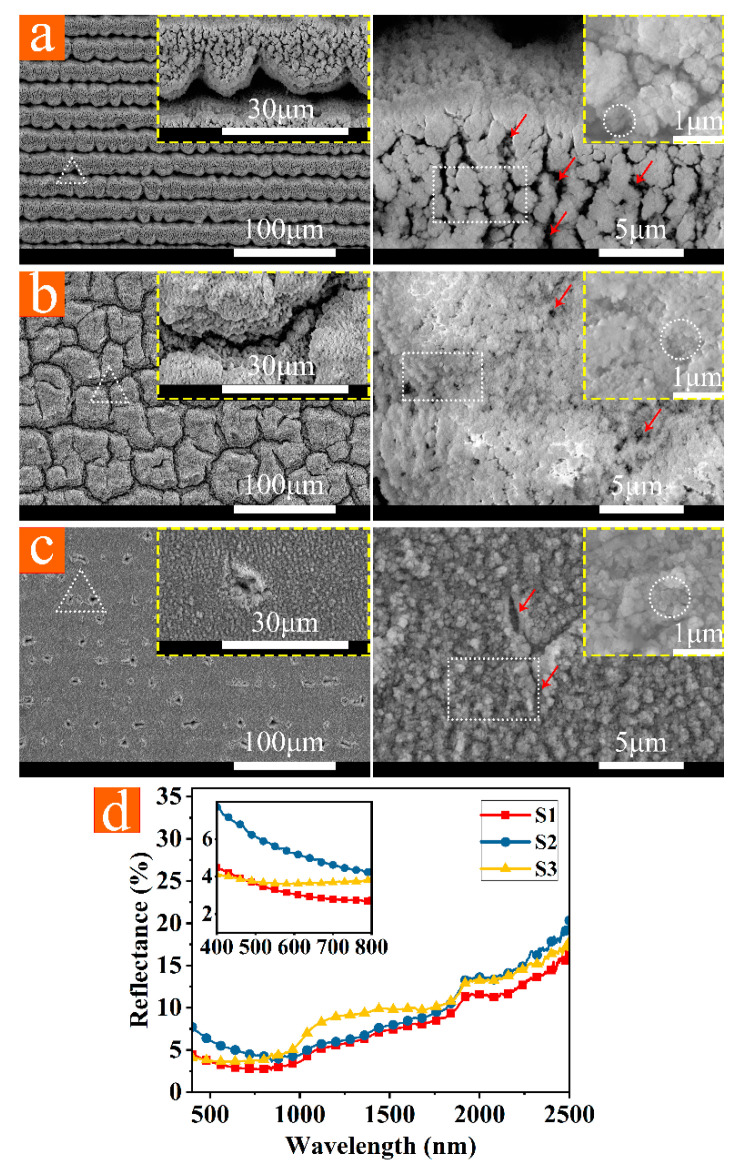
(**a**–**c**) SEM photographs (magnification of 1200 times, 5000 times, 20,000 times, and 100,000 times) of S1, S2, and S3. (**d**) Reflectance curves of S1–S3. Triangles (white), rectangles (white), and circles (white) represent, respectively, microscale light-trapping structures, subwavelength structures, and nanoparticles.

Microscale light-trapping structures, subwavelength structures, and nanoparticles are visible on SEM images with scales of 100 μm, 5 μm, and 1 μm. The blackened aluminum alloy was made via a sizeable defocusing laser beam with high power. As observed in Figure 4d, the average reflectance of S1–S3 (Figure 4a–c) is below 10% within the wavelength range of 400–2500 nm. Under the magnification of 20,000 times and 100,000 times, there are plentiful subwavelength structures and nanoparticles on the surfaces of S1–S3. The blackening was caused by multi-scattering attributable to the aggregated nanoparticles [35]. The surface particles of S2 are smaller and flocculent, which is not as various as S1 and S3; thus, its reflectivity is higher in the visible spectral region (400–800 nm). The shallow structures of S1–S3 cannot achieve effective light-trapping effects for long electromagnetic waves. Thus, reflectance increases significantly with increases in incident wavelength. The refractive indices of the effective medium layers are estimated [13] by n_eff_ = fn_metal_ + (1−f)n_air_, and f is a filling factor of metal in the range 0–1. The more porous and rougher surfaces have smaller filling factors, and as f becomes smaller, surface reflectance is reduced gradually [13]. Structures in Figure 4a have more pores (as shown in red arrows) than in Figure 4b,c; thus, the reflectance curve of S1 moves down relative to S2 and S3.

### 3.3. Antireflection Structure in the Vis-NIR Spectral Region: Microscale Light-Trapping Structure + Subwavelength Structure + Nanoparticle

**Figure 5:**
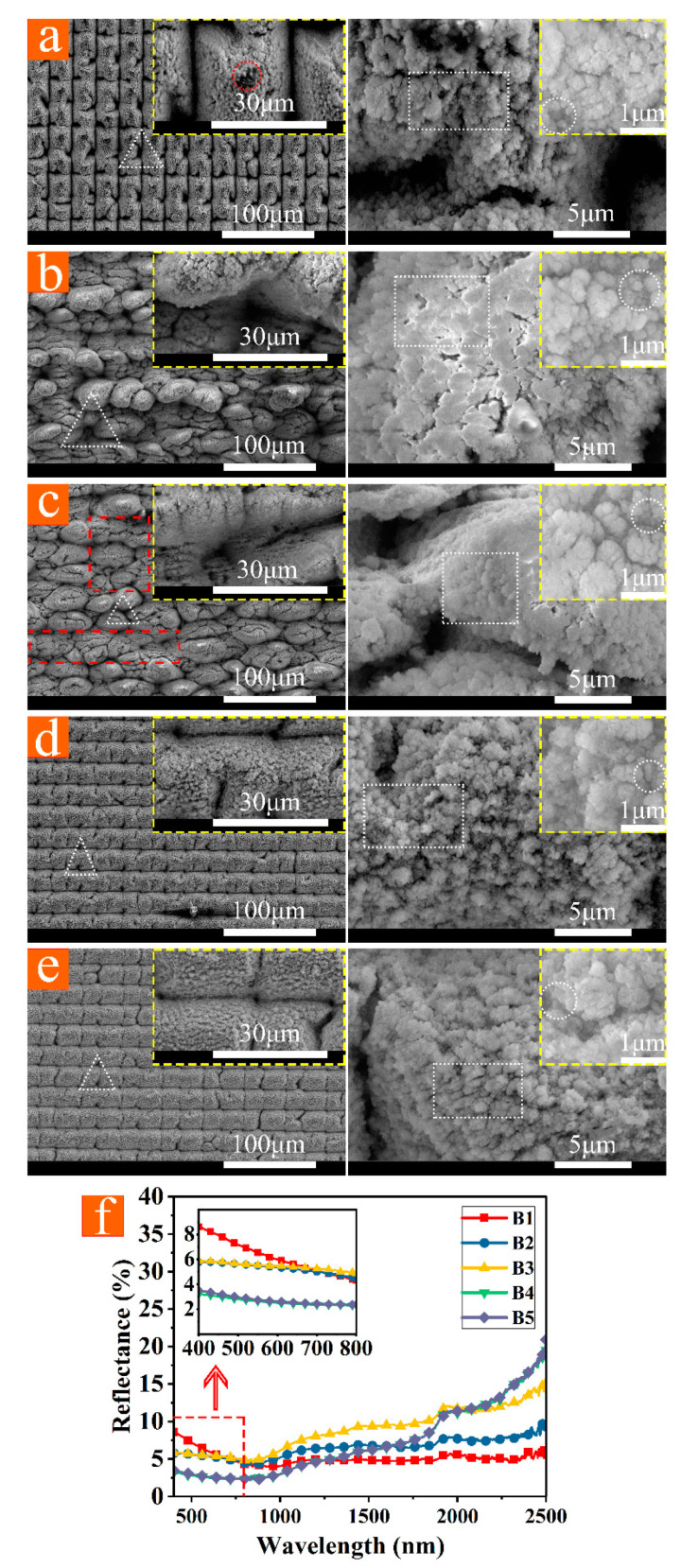
(**a**–**e**) SEM photographs (magnification of 1200 times, 5000 times, 20,000 times, and 100,000 times) of B1, B2, B3, B4, and B5. (**f**) Reflectance curves of B1–B5. Triangles (white), rectangles (white), and circles (white) represent, respectively, microscale light-trapping structures, subwavelength structures, and nanoparticles.

It is necessary to have microscale light-trapping structures, subwavelength structures, and abundant nanoparticles simultaneously in order to achieve high-performance antireflection effects in a wide wavelength range. Thus, the laser processing parameters were improved based on the S series, as shown in Figure 1. In our experiment, the blackened parameters of the polished surface were also applicable to samples with microscale structures. B1–B5 (Figure 5a–e) micro-nano structures were prepared by multiple fast laser scans with low power and a single slow laser scan with high power and large defocusing. Among them, the power of the slow laser scan of B2 and B3 was higher (60 W); thus, the heat-affected zones of laser ablation were more extensive. As a result, melting and recasting phenomena were prominent, causing the structures to be more irregular [36]. Compared with the relatively flat surfaces of B4 and B5, the structure of B1 has a regularly arranged pointed top, as shown by the red circle in Figure 5a. The surface of B3 has flat structures with inconspicuous height fluctuations, as shown by the red rectangular in Figure 5c. In contrast, B1 has the best light-trapping effect. The light-trapping structures of B4 and B5 are shallow without obvious wide micron gaps; thus, reflectance increases significantly with increasing wavelength. However, their average reflectance is kept below 3% from 400 nm to 800 nm because of abundant nanoparticles. The microscale structures of B1 have a relatively sizeable depth-diameter ratio with obvious wide micron gaps. Structure B1 has hierarchical structures (illustrated in Figure 2) with multiple antireflection mechanisms; thus, the average reflectance is 5.14% from 400 nm to 2500 nm.

**Figure 6:**
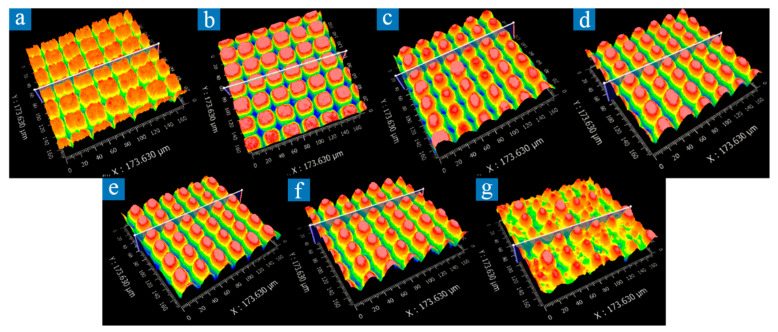
Three-dimensional topography of aluminum alloy surface captured by a white light interferometer under different laser power. Length in the images is 173.63 μm. (**a**) 27 W; (**b**) 28.8 W; (**c**) 30.6 W; (**d**) 32.4 W; (**e**) 34.2 W; (**f**) 36 W; (**g**) 37.8 W.

The speed of a laser scan when processing structure B was selected according to the previous experimental experience. During the experiment, it was observed that the laser power and scanning intervals have a significant impact on the microscale frame structures. Thus, the single variable experiments of power and scanning intervals were performed, respectively. The processing target of the microscale frame structure is conical. It is defined that the regular topography conforming to the ideal structure (conical) is of good quality. The processing effect we expect is shown in Figure 6d–f. By using 3D topography observations in Figure 6, some observations can be made. The higher the laser power is, the higher the laser fluence is. When laser fluence is higher than the ablation threshold of aluminum alloy, the aluminum alloy will be melted and recast or even gasified by the femtosecond pulse with high peak power. The laser energy obeys Gaussian distribution. The energy density of the central part first reaches the ablation threshold, and the periphery slowly reaches the ablation threshold [37], increasing the transverse dimension of the etched structure. Secondly, when the power becomes too large, laser energy becomes unstable, and the ablation quality of the microscale structures at high energy is poorer than that at low energy [36]. The physical-chemical reactions on the metal surface are more complex and intense, resulting in the actual processed structure deviating from the expectation, as shown in Figure 6g. Therefore, mild laser power (32.4~36 W) benefits the quality and morphology control of the processed microscale structures, as shown in Figure 6d–f.

**Figure 7:**
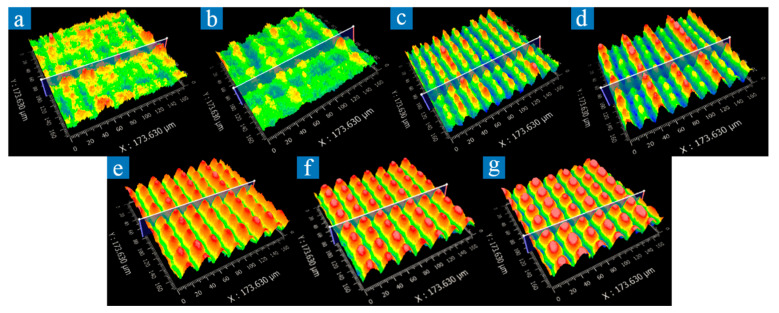
Three-dimensional topography of aluminum alloy surface captured by a white light interferometer under different scanning interval. Length in the images is 173.63 μm. (**a**) 14 μm; (**b**) 16 μm; (**c**) 18 μm; (**d**) 20 μm; (**e**) 22 μm; (**f**) 24 μm; (**g**) 26 μm.

As shown in Figure 7a–d, when the scanning interval is too small, the scanning path of the laser beam is partially overlapped and cannot obtain regular microscale structures. An excessive scanning interval results in a decrease in the duty ratio of microscale structures from 1 to less than 1, leaving an original surface and reducing antireflection performance [8,38].

## 4. Conclusions

The femtosecond laser is used to fabricate micro/nanostructures for antireflection on the surface of 2A12 aluminum alloy. Different micro-nano structures and antireflection properties can be obtained by adjusting laser processing parameters. A slow laser scan with high power and sizeable defocusing can prepare blackened aluminum alloy. The blackened parameters of the polished surface are also applicable for samples with specific microscale frame structures. Subwavelength structures and nanoparticles can reduce the reflectance of aluminum alloy in the visible light band by exciting plasmon resonance and tuning the optical response of the surface. Microscale light-trapping structures, subwavelength structures, and nanoparticles can reduce the reflectance of aluminum alloy in the Vis-NIR spectral region by enhancing the light-trapping effect, exciting plasmon resonance, and tuning the optical response of the surface. The light-trapping effect of microscale frame structures can effectively inhibit the increase in reflectance of the antireflection micro/nanostructures on the aluminum alloy surface as the wavelength increases. The hierarchical structures formed by the microscale structures, subwavelength structures, and the rich nanoparticles obtained by the processing with blackened parameters can yield excellent antireflective properties in a broad spectrum. About 97.4% absorptivity in the visible spectral region (400–800 nm) and 94.8% absorptivity over the Vis-NIR spectral region (400–2500 nm) can be achieved. The method of processing micro-nano structures described in this article has the advantages of low cost, high efficiency, and strong adaptability.

## Figures and Tables

**Table 1:** Chemical composition of the 2A12 aluminum alloy (%).

Al	Si	Fe	Cu	Mn	Mg	Cr	Zn	Ti	Others
≥92.87	0.08	0.26	4.42	0.49	1.58	0.01	0.12	0.02	≤0.15

**Table 2:** Processing parameters for different samples.

Samples	Pulse Energy (μJ)	Power (W)	Fluence (J/cm^2^)	Velocity (mm/s)	Interval (mm)	Repeat	Defocusing (mm)
S1	84	42	18.11	20	22	5	4
S2	96	48	20.70	10	10	3	3.8
S3	84	42	18.11	20	10	1	4.5
B1	60	30	12.94	800	25	80	0
	96	48	20.70	30	25	1	4
B2	60	30	12.94	800	25	100	0
	120	60	25.87	25	25	1	4.5
B3	60	30	12.94	800	25	100	0
	120	60	25.87	50	25	1	4.5
B4	62.4	31.2	13.45	800	23	120	0
	84	42	18.11	25	23	1	4
B5	62.4	31.2	13.45	1000	22	120	0
	84	42	18.11	25	22	1	4
